# Gut-brain axis: gut dysbiosis and psychiatric disorders in Alzheimer’s and Parkinson’s disease

**DOI:** 10.3389/fnins.2023.1268419

**Published:** 2023-11-13

**Authors:** Charlotte R. Denman, Sang Myun Park, Junghyun Jo

**Affiliations:** ^1^Okinawa Institute of Science and Technology Graduate University, Okinawa, Japan; ^2^Department of Pharmacology, Ajou University School of Medicine, Suwon, Republic of Korea; ^3^Center for Convergence Research of Neurological Disorders, Ajou University School of Medicine, Suwon, Republic of Korea; ^4^Neuroscience Graduate Program, Department of Biomedical Sciences, Ajou University School of Medicine, Suwon, Republic of Korea

**Keywords:** gut-brain axis, gut microbiome, Alzheimer’s disease, Parkinson’s disease, anxiety, depression

## Abstract

Gut dysbiosis and psychiatric symptoms are common early manifestations of Alzheimer’s disease (AD) and Parkinson’s disease (PD). These diseases, characterised by progressive neuron loss and pathological protein accumulation, impose debilitating effects on patients. Recently, these pathological proteins have been linked with gut dysbiosis and psychiatric disorders. The gut-brain axis links the enteric and central nervous systems, acting as a bidirectional communication pathway to influence brain function and behavior. The relationship triad between gut dysbiosis, psychiatric disorders, and neurodegeneration has been investigated in pairs; however, evidence suggests that they are all interrelated and a deeper understanding is required to unravel the nuances of neurodegenerative diseases. Therefore, this review aims to summarise the current literature on the roles of gut dysbiosis and psychiatric disorders in pathological protein-related neurodegenerative diseases. We discussed how changes in the gut environment can influence the development of psychiatric symptoms and the progression of neurodegeneration and how these features overlap in AD and PD. Moreover, research on the interplay between gut dysbiosis, psychiatric disorders, and neurodegeneration remains in its early phase. In this review, we highlighted potential therapeutic approaches aimed at mitigating gastrointestinal problems and psychiatric disorders to alter the rate of neurodegeneration. Further research to assess the molecular mechanisms underlying AD and PD pathogenesis remains crucial for developing more effective treatments and achieving earlier diagnoses. Moreover, exploring non-invasive, early preventive measures and interventions is a relatively unexplored but important avenue of research in neurodegenerative diseases.

## 1. Introduction

Neurodegenerative diseases are a class of disorders characterised by the progressive loss of neurons in the central nervous system (CNS) that are progressive in nature (Agnello and Ciaccio, 2022). Most neurodegenerative diseases are sporadic with no clear causes; although certain neurodegenerative diseases are known to be inherited, aging is a predominant risk factor. Other risk factors include genetic abnormalities, lifestyle factors, and environmental factors. Therefore, a growing and aging population is expected to have a severe impact on the number of affected individuals, with a drastic increase in disease prevalence by 2050 (Dorsey et al., 2018; Nichols et al., 2022). A predicted increase in cases of 2.6-fold for Alzheimer’s disease (AD) and 2-fold for Parkinson’s disease (PD) by 2050 and 2030, respectively, is expected to have a large socioeconomic burden. Neurodegenerative diseases are often diagnosed upon the development of obvious symptoms that tend to manifest years, post-onset (DeTure and Dickson, 2019; Raza et al., 2019). AD and PD are the two most common neurodegenerative diseases, with 55 million and 6 million cases globally at present, respectively.

AD is a form of dementia in which neurodegeneration initially occurs in the entorhinal cortex and hippocampus, progressing to other areas of the cerebral cortex (DeTure and Dickson, 2019); conversely, PD is clinically characterised as a motor disorder by loss of dopaminergic neurons in the substantia nigra pars compacta (Raza et al., 2019). The symptoms of these diseases reflect the area in which neuronal loss occurs. Memory deficits, confusion, impaired judgment, and language deficits are characteristic of AD, whereas tremors, bradykinesia, muscle rigidity, and impaired balance are typical of patients with PD. These symptoms have severe and lasting impacts not only on patients, but also on families, friends, and caregivers.

**Figure 1:**
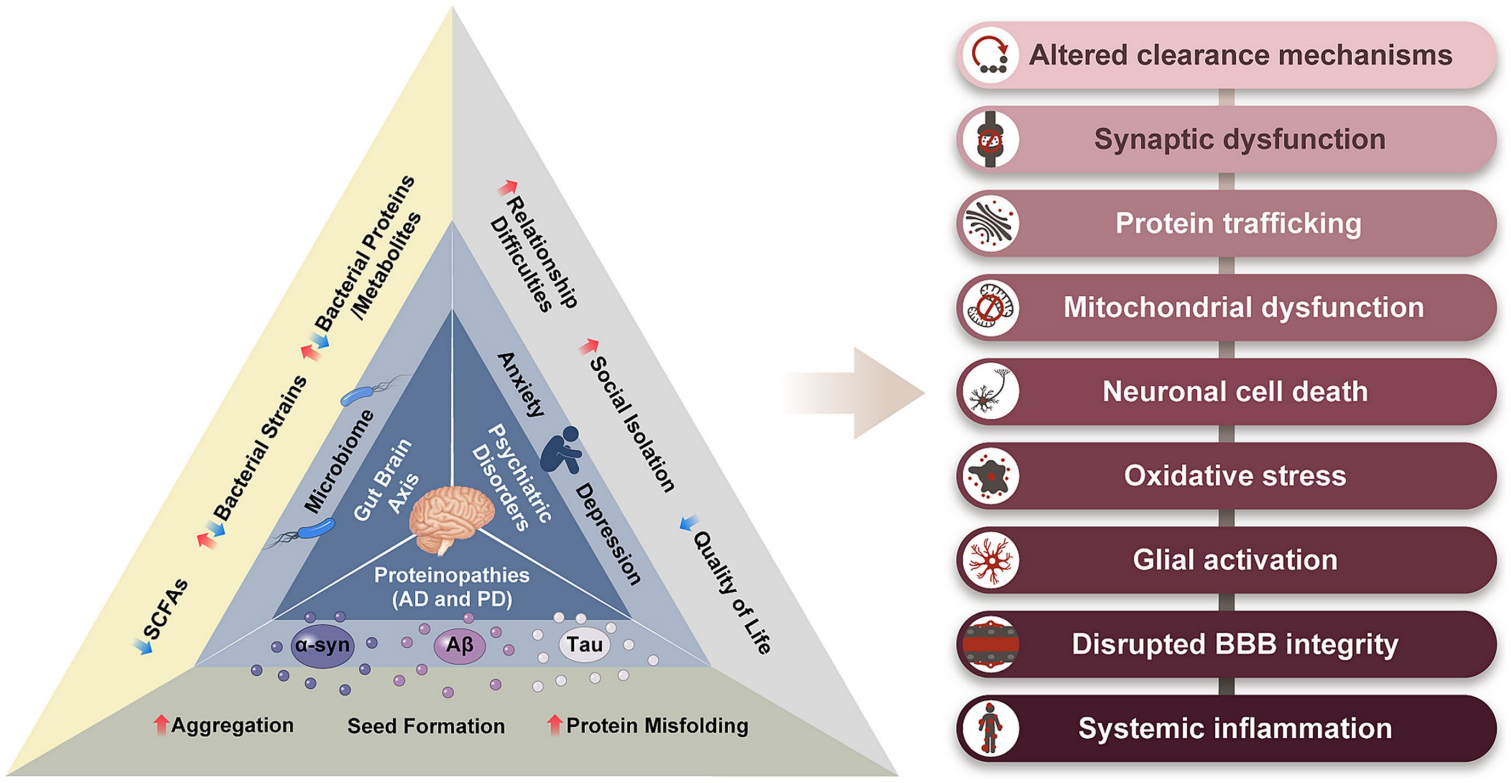
The relationship triad between proteinopathy, gut-brain axis, and psychiatric disorders, in AD and PD. The common symptoms resulting from these three features of AD and PD are listed on the right. α-syn, alpha-synuclein; Aβ, amyloid beta; SCFs, short chain fatty acids.

Although there are distinct differences between patients with AD and PD, there are broad similarities. Proteinopathy, neuroinflammation, oxidative stress, blood brain barrier (BBB) disruption, neurotransmitter depletion, and neuronal death are common features of AD and PD (Dugger and Dickson, 2017). Furthermore, emerging evidence indicates alterations in the gut microbiome of patients with AD and PD, acting via the gut-brain axis (GBA), prior to the onset of characteristic symptoms. Psychiatric symptoms, such as depression and anxiety, have also been reported to precede the appearance of characteristic symptoms in AD and PD, and notably are also linked with the GBA (Zhang et al., 2022a; Intili et al., 2023; Pagonabarraga et al., 2023). The treatment of these disorders is currently limited to symptom management. There is an urgent need to develop preventive measures and disease-modifying treatments. The aim of this review is to provide a broader perspective on AD and PD by exploring some of their overlapping features, particularly the gut microbiome and psychiatric disorders. By adopting a broader perspective, we can uncover important clues pertaining to the causes of neurodegeneration and prompt new avenues for therapeutic exploration (Figure 1).

## 2. The gut-brain axis

The GBA is a bidirectional communication pathway between the gastrointestinal tract and brain (Mayer, 2011), which encompasses several different entities, including the vagus nerve, a part of the autonomic nervous system, as well as the endocrine system, immune system, hypothalamic–pituitary–adrenal axis, autonomic nervous system, enteric nervous system, CNS, and gut microbiota (Cryan and Dinan, 2012).

Braak and colleagues initially postulated the involvement of the GBA in PD in the late 90s; they also hypothesised that the pathological protein, alpha-synuclein (α-syn), propagates in a spatiotemporal manner via a defined route to and throughout the CNS (Braak et al., 2003). The GBA was not originally proposed by Braak when describing the spatiotemporal manner of amyloid beta (Aβ) where propagation was thought to begin in the entorhinal cortex (Braak and Braak, 1991). However, more recent studies have implicated the GBA’s involvement in the transmission Aβ from the gut to the CNS (Sun Y. et al., 2020). The pathological forms of Aβ and α-syn are hallmark features of AD and PD, respectively, and endow these diseases as proteinopathies (Hetz and Saxena, 2017). These proteins misfold, aggregate, and provide templates for further aggregation, thereby creating a harmful feed-forward loop. Meanwhile, tau is also common in AD and PD; however, the evidence so far suggests that it is controversial whether tau is associated with the enteric and CNS pathologies, as has been observed for Aβ and α-syn (Derkinderen et al., 2021). Despite the presence of distinct protein aggregates as characteristic markers of these diseases, it is likely that multiple protein interactions may be responsible for conveying crucial information in AD and PD. A previous review by Sengupta and Kayed investigated the crosstalk between the three most noted pathological proteins, Aβ, α-syn, and tau, and highlighted the importance of a personalised combination approach when searching for new therapeutic strategies (Sengupta and Kayed, 2022). By discerning the diverse array of protein aggregates exhibited by the patient and using a synergistic blend of immunotherapies to specifically target these aggregates, this highly personalised therapeutic approach has the potential to yield profound clinical advantages. Although tau may not align with Braak’s hypothesis in the same manner as Aβ and α-syn, the potential role of GBA in AD and PD has attracted significant attention (Rietdijk et al., 2017). In particular, the gut microbiome has become a central focus in the GBA story of neurodegeneration (Morais et al., 2021).

The gut is host to trillions of microorganisms, which are collectively referred to as the gut microbiome (Cryan et al., 2020); this large microflora ecosystem plays a major role in maintaining the physiological balance of human health. Unique to each individual, factors such as the birth mother’s gut microbiome, geographical environment, diet, and medications influence its composition (Morais et al., 2021). The gut microbiome plays a crucial role in multiple physiological processes, including digestion, hormone regulation, and neurotransmitter release (Mayer, 2011). In the context of digestion, the gut microbiota effectively ferment indigestible substrates, secrete enzymes, and synthesise essential vitamins and nutrients, thereby having a significant impact on the overall health of an individual (Gomaa, 2020). With regards to hormone regulation, the gut microbiome is extensively involved in hormone synthesis, metabolism, and signalling (Martin et al., 2019). This interaction between the gut microbiota and hormones is known as the gut-brain-endocrine axis, which involves complex communication pathways between the gut, the brain, and the endocrine system. Moreover, the gut microbiota assumes a pivotal role in neurotransmitter release, particularly in serotonin synthesis, where over 90% of serotonin production occurs in the gut (Ge et al., 2017). Additionally, these bacteria possess the capability to synthesise other neurotransmitters like dopamine, GABA, and noradrenaline, while also influencing the availability of neurotransmitter precursors (Strandwitz, 2018).

Dysbiosis is characterised by an imbalance in the gut microbiome’s composition, marked by an overabundance of harmful or pathogenic microorganisms, a reduction in beneficial bacteria, or an overall decline in microbial diversity within the gut (Lloyd-Price et al., 2016). This perturbation in gut microbiota can significantly impact health and is associated with a diverse array of pathological conditions, including neurodegenerative and neuropsychiatric disorders (Konjevod et al., 2021; Mitrea et al., 2022; Basiji et al., 2023). Although not covered in this review, it is crucial to acknowledge that sex differences within the gut-brain axis could impact the development and progression of neurological disorders and treatment approaches, and we recommend referring to Holingue et al. (2020) comprehensive review for a detailed exploration of this subject. Remarkably however, gastrointestinal discomfort and mental health disorders are frequently reported symptoms in both AD and PD (Zhang et al., 2022a; Intili et al., 2023). This compelling correlation has inspired a new wave of research, as investigations delve into unravelling the potential connections linking the gut microbiome, psychiatric symptoms, and the underlying processes of neurodegeneration.

## 3. The gut microbiome in AD and PD

Studies involving humans, mice, and *in vitro* models have provided insights into the involvement of the gut microbiome in AD and PD (Scheperjans et al., 2015; Vogt et al., 2017; Ho et al., 2018; Seo et al., 2023). Several bacterial strains are differentially expressed in patients with neurodegenerative disorders compared with healthy age-matched controls (Romano et al., 2021; Chandra et al., 2023). Further investigations of these bacterial strains have identified links to various pathological mechanisms involved in neurodegeneration, including protein aggregation and neuroinflammation. Research has uncovered alterations in the gut microbiota of patients with neurodegenerative diseases, with evidence suggesting that bacterial proteins and metabolites play key roles.

### 3.1. Bacterial proteins

Recently, bacterial proteins have emerged as intriguing players in the complex landscape of neurodegenerative diseases. One prominent example is the bacterial amyloid protein known as curli, which shares structural similarities with the Aβ protein implicated in AD (Chapman et al., 2002). Researchers have found evidence suggesting that curli proteins can cross-seed Aβ aggregation, hastening the formation of toxic amyloid aggregates in the brain (Friedland et al., 2020). This cross-seeding mechanism implies that curli may serve as a catalyst, effectively initiating the aggregation of host proteins involved in neurodegeneration. Furthermore, the role of curli in PD has been investigated. Utilising animal models, researchers have demonstrated that oral exposure to curli-producing bacteria results in elevated aggregation of α-syn coupled with the development of motor symptoms. Administering a chemical modulator that inhibits curli production prevents these pathological events, underscoring the potential involvement of curli in PD pathogenesis (Chen et al., 2016; Sampson et al., 2020; Wang C. et al., 2021). Recent metagenomic profiling of patients with PD compared with healthy age-matched controls has reinforced these findings, revealing the upregulation of several curli genes in PD cases, substantiating the outcomes observed in animal models (Wallen et al., 2022).

Curli has received attention not only for its potential role in protein aggregation, but also for its neuroinflammatory effects. When curli proteins interact with the CNS, they can stimulate microglia activation, the resident immune cells of the brain (Chen et al., 2016). This activation triggers a pro-inflammatory response characterised by the release of cytokines and other inflammatory molecules. Prolonged and dysregulated neuroinflammation is a common feature of various neurodegenerative diseases, including AD and PD, contributing to neuronal damage and dysfunction (Zhang W. et al., 2023). Therefore, the ability of curli to induce neuroinflammation further highlights its multifaceted effect on the pathogenesis and progression of neurodegenerative diseases, making it a subject of intense research interest. Alternatively, curli in the gut might prime immune cells and lead to heightened responses to amyloid proteins within the brain (Chen et al., 2016; Miraglia and Colla, 2019). Curli is not an isolated instance, as various bacteria within the gut can produce amyloid-like proteins. Their cumulative effect may be additive, or alternatively, these diverse bacterial species may influence the pathogenesis of distinct neurodegenerative diseases (Miraglia and Colla, 2019; Friedland et al., 2020; Wang and Zheng, 2022). Despite the intriguing observations linking curli to the exacerbation of protein aggregation, our understanding of the underlying mechanisms and whether this relationship is causative or consequential remains limited.

While curli proteins have gained attention for their potential involvement in neurodegenerative diseases, other bacterial proteins have also been explored in this context. For instance, bacterial enzymes such as proteases have also been shown to influence the aggregation of misfolded proteins, such as tau in AD or α-syn in PD (Dominy et al., 2019; Feng et al., 2020; Nonaka and Nakanishi, 2020; Chi et al., 2021). *Porphyromonas gingivalis* (*P. gingivalis*), a gram-negative bacterium primarily associated with periodontal disease, has recently come into focus for its potential involvement in neurodegenerative diseases, particularly AD. One of the key findings is the presence of gingipain, a protease from *P. gingivalis*, in the brains of patients with AD. Gingipain can cleave host proteins, including those involved in maintaining neuronal cells and preventing the accumulation of toxic protein aggregates, such as Aβ (Dominy et al., 2019). The presence of gingipain in the brain suggests a potential mechanism by which *P. gingivalis* may contribute to AD pathogenesis. Furthermore, studies using animal models have provided experimental evidence supporting the connection between *P. gingivalis* and neurodegenerative diseases (Feng et al., 2020; Chi et al., 2021). These studies have shown that oral infection with *P. gingivalis* can lead to Aβ accumulation in the brain, cognitive impairments, and neuroinflammation. Furthermore, *P. gingivalis* was accompanied by alterations in gut homeostasis. Further research is needed to establish a definitive causal relationship and understand to what extent it may act through the GBA. Nevertheless, the emerging evidence linking gingipains to neurodegenerative diseases, particularly AD, is intriguing.

### 3.2. Bacterial metabolites

Metabolites of the gut microbiota play an important role as signalling messengers between the gut microorganisms and host. Among these metabolites, short-chain fatty acids (SCFAs), *p*-cresol, and indoles serve crucial physiological functions. An imbalance in these metabolites has been implicated in the pathogenesis of several diseases (Lavelle and Sokol, 2020; Liu et al., 2022).

#### 3.2.1. Short chain fatty acids

SCFAs originate from the fermentation of dietary fibers by gut bacteria during digestion; hence, SCFA type depends on the ingested fiber. The most common SCFAs in the gut of humans are butyric, acetic, and propionic acids (Xiong et al., 2022). SCFAs have been associated with various neuroprotective roles, although research in this area is still in its infancy (Silva et al., 2020). Butyrate has demonstrated anti-inflammatory properties by suppressing the activation of microglial cells and reducing the production of pro-inflammatory cytokines in the brain (Wenzel et al., 2020; Liu et al., 2021; Caetano-Silva et al., 2023). Furthermore, SCFAs have been identified as inhibitors of histone deacetylase enzymes, leading to increased histone acetylation. This epigenetic modification can enhance the expression of genes associated with synaptic plasticity and neuronal survival (Patnala et al., 2017; Caetano-Silva et al., 2023). Some studies have also suggested that SCFAs may stimulate the release of neurotrophic factors, such as brain-derived neurotrophic factors, promoting the survival, growth, and maintenance of neurons (Barichello et al., 2015; Varela et al., 2015). SCFAs also influence the BBB by enhancing the expression and assembly of tight junction proteins in endothelial cells (Braniste et al., 2014). Tight junctions are critical for sealing the gaps between these cells, forming a tight barrier that restricts the entry of molecules and pathogens into the brain.

Recent interest in SCFAs in neurodegenerative diseases has stemmed from the observation that compared with healthy controls, individuals with AD and PD have reduced SCFA levels in their faecal microbiome, and that these levels correlate with disease severity (Unger et al., 2016; Aho et al., 2021; Baert et al., 2021; Wu et al., 2021). An interesting study by Sampson et al. demonstrated that introducing microbiota from patients with PD to α-syn overexpressing mice exacerbated motor symptoms compared with microbiota transplants from healthy human donors (Sampson et al., 2016). Oral administration of SCFAs can ameliorate symptoms of neurodegeneration, possibly acting via glucagon-like peptide-1 (Govindarajan et al., 2011; Liu et al., 2017). However, previous studies have investigated this relationship and discovered that SCFAs contribute to disease pathogenesis (Sun et al., 2018; Colombo et al., 2021). These discrepancies are possibly attributed to different SCFA doses, sampling measures, and models. Despite the inconsistencies, these studies have highlighted the involvement of the gut microbiome, and more widely, the GBA, in neurodegeneration. These discoveries expand our understanding of these diseases and facilitate the development of new treatments.

#### 3.2.2. *p*-Cresol and *p*-cresol sulphate

*p*-Cresol is a microbial metabolite produced by the gut microbiota during the digestion of dietary compounds, particularly tyrosine and phenylalanine. *p*-Cresol can undergo secondary metabolism, primarily in the liver, where it is conjugated with a sulphate group, giving rise to *p*-cresol sulphate, a protein-bound uremic toxin (UT) (Liabeuf et al., 2011). Elevated levels of *p*-cresol sulphate have been associated with several diseases and health implications, including chronic kidney disease, cardiovascular disease, gastrointestinal disorders, bone health, and neurological and cognitive impairment (Liu et al., 2018; Lin et al., 2019). It is important to emphasise that the link between *p*-cresol sulphate and these health complications is primarily observed in the context of chronic kidney disease and related kidney disorders (Liu et al., 2018). In individuals with healthy renal function, the body efficiently processes and eliminates *p*-cresol sulphate, thus diminishing its potential impact on health (Gryp et al., 2017). Nevertheless, the influence of protein-bound UTs on AD and PD is an emerging area of research that merits examination of their role in these conditions.

A study revealed elevated levels of *p*-cresol sulphate in the cerebrospinal fluid (CSF) of patients with PD compared with healthy age-matched controls (Sankowski et al., 2020). None of these PD patients had symptoms of chronic kidney disease, although the PD group had slightly lower eGFR, an indicator of kidney function, compared to the control group. Despite a lower eGFR, the level reported is only just below the lower limit of normal, 90 mL/min/1.73m^2^ (Tarwater, 2011). Furthermore, eGFR was calculated using the abbreviated Modification of Diet in Renal Disease (MDRD) equation, which is a widely recognised method. However, it is important to note that this method is an estimation and is reportedly unreliable when eGFR exceeds 60 mL/min/1.73m^2^ (Tarwater, 2011). Nevertheless, it is still possible that kidney function might be lower in individuals with PD, which could contribute to elevated *p*-cresol sulphate levels, as suggested by Sankowski et al. (2020). However, alternative mechanisms such as increased gut permeability or imbalances in the gut microbiota of PD patients may explain the observed increase in *p*-cresol sulphate levels. Bacteria from the *Lactobacillaceae* and *Bifidobacteriaceae* families are known to produce *p*-cresol, and elevated levels of these bacteria have been reported in PD individuals (Saito et al., 2018; Romano et al., 2021). Therefore, it is plausible that the higher levels of *p*-cresol sulphate in the CSF of PD patients may result from increased populations of *p*-cresol-producing bacteria. Furthermore, increased gut permeability is a characteristic observed in chronic kidney disease patients, which is believed to facilitate the circulation of UTs (Cosola et al., 2021). Heightened gut permeability is also observed in PD patients (Clairembault et al., 2015). Consequently, alterations in gut microbiota and/or increased gut permeability may contribute to the elevated CSF levels of *p*-cresol sulphate in individuals with PD. These effects could occur independently, in conjunction with reduced kidney function, or through other mechanisms that are presently unknown.

An *in vivo* study found that exogenous administration of *p*-cresol sulphate to mice resulted in cognitive impairment, increased oxidative stress, neuroinflammation, and decreased brain-derived neurotrophic factor levels. The authors also observed anxiety-and depressive-like behaviors in the mice exposed to *p*-cresol sulphate, which are recognised symptoms in AD and PD (Sun C. Y. et al., 2020). Another study revealed decreased dopaminergic neuron excitability in the ventral tegmental area of mice exposed to *p*-cresol, which was rescued by faecal microbiota transplantation (FMT) from control mice (Bermudez-Martin et al., 2021). This finding highlights the potential role for the gut microbiota in altered levels of *p*-cresol, and consequently *p*-cresol sulphate, as opposed to kidney function impairment. Furthermore, an *in vitro* study using the N2a and PC12 cell line, commonly used in AD research, found *p*-cresol adversely affects dendrite development, synaptogenesis, synaptic function, and oligodendrocyte function (Guzman-Salas et al., 2022; Needham et al., 2022; Xie et al., 2022). However, the connection between *p*-cresol and AD remains relatively unexplored in comparison to PD and requires investigation.

Overall, *p*-cresol sulphate has demonstrated the ability to induce inflammation and oxidative stress, both characteristic features of neurodegenerative diseases. Furthermore, *p*-cresol sulphate has been linked to anxiety, depression, reduced neuron excitability, and oligodendrocyte impairment. These studies encompassed patients without chronic kidney disease and *in vitro* cultures, demonstrating the involvement of *p*-cresol and *p*-cresol sulphate in neurodegenerative disorders. As mentioned above, increased levels of *p*-cresol and *p*-cresol sulphate in individuals with neurodegenerative conditions could be attributed to diminished kidney function, increases in *p*-cresol-producing bacteria, a combination of both, or an alternative unknown mechanism. Interestingly, an FMT was able to rescue the neurotoxic effects of *p*-cresol, highlighting a promising therapeutic approach in AD and PD patients with elevated protein-bound UT levels. It is critical that future research continues to delve into the mechanisms responsible for the elevation of *p*-cresol and *p*-cresol sulphate levels to guide therapeutic interventions aimed at these metabolites.

#### 3.2.3. Indoles and indoxyl sulphate

Tryptophan, a dietary amino acid, undergoes digestion by gut microbes, resulting in the production of indole and its derivatives. The specific derivatives generated depend on the bacteria involved in the digestion process. Similar to *p-*cresol, indole can also undergo secondary metabolism in the liver, being converted to indoxyl-sulphate, another uremic toxin (Liabeuf et al., 2011). Altered levels of indoles and their derivatives and secondary metabolites in AD and PD have prompted research into their roles in the development and pathogenesis of these diseases (Wu et al., 2021; Chen et al., 2022). In the same study by Sankowski and colleagues that found increased levels of *p*-cresol sulphate in the CSF of PD patients, indoxyl-sulphate was also found to be increased. Elevated levels of these UTs in the CSF were associated with biomarkers of inflammation and oxidative stress, as well as motor fluctuations in PD individuals (Sankowski et al., 2020). Cognitive impairment is a recognised symptom among patients with chronic kidney failure. One study reported that treatment of indoxyl-sulphate to astrocyte and neuron cultures resulted in an increase in reactive oxygen species and increased cell death, respectively (Adesso et al., 2017). This outcome suggests a potential connection between elevated indoxyl-sulphate levels and cognitive dysfunction. Moreover, the use of AST-120, an indoxyl-sulphate adsorbent, was found to reduce inflammatory responses in astrocytes exposed to serum from chronic kidney failure patients (Adesso et al., 2018). This study underscores the therapeutic promise of targeting indoxyl-sulphate to alleviate inflammation and warrants further investigation, particularly within in the context of neurodegenerative diseases. A noteworthy study conducted by Karbowska et al. (2020) investigated the effects of indoxyl-sulphate treatment in rats, focusing on neurotransmitter levels and behavioral changes. They observed that indoxyl-sulphate could accumulate within the brain, primarily in the brainstem, but also in the striatum with hippocampus, cerebellum, and cortex regions. They detected reduced levels of noradrenaline, dopamine, and serotonin following treatment with indoxyl-sulphate and these changes coincided with alterations in locomotion and mood, characteristic features seen in PD. These findings provide evidence for a role that indoxyl-sulphate may play in the pathogenesis of neurodegenerative disorders, such as AD and PD.

Research into indoxyl-sulphate’s precursor, indole, in neurodegeneration has also been investigated. Two indole derivatives, indole-3-propionic acid and indole-3-pyruvic acid have emerged as predictive factors for AD progression when measured in plasma and faecal samples, respectively (Huang Y. L. et al., 2021; Wu et al., 2021). Indole derivatives exhibit varying expression patterns, with upregulation and downregulation observed depending on the derivative. For instance, indole-3-propionic acid displayed elevated levels in AD and PD, whereas 5-hydroxyindole, indole-2-carboxylic acid, and 3-(2-hydroxyethyl) indole exhibited downregulated levels (Huang Y. L. et al., 2021; Wu et al., 2021; Chen et al., 2022). These observations have prompted preclinical investigations using animal models to explore the mechanisms through which indole and its derivatives influence neurodegeneration. In an APP/PS1 mouse model, a combination of indole-3-propionic acid and indole-3-acetic acid was discovered to mitigate microglial activation and decrease the expression of inflammatory cytokines. These effects were associated with their ability to upregulate the aryl hydrocarbon receptor and inhibit the NF-κB signalling pathway (Sun et al., 2022). Although prior findings indicate elevated levels of indole-3-propionic acid in AD and PD (Huang Y. L. et al., 2021, Chen et al., 2022), this study administered a combination of indole derivatives, including indole-3-propionic acid. We have considered several possible explanations for the discrepancy between finding elevated levels of indole-3-propionic acid in AD and PD patients and using indole-3-propionic acid as a therapeutic strategy. Given that a combination of indole derivatives was administered in this study, attributing the observed neuroprotective effects to a particular indole becomes challenging. Furthermore, the positive effect of indole-3-acetic acid may have outweighed any potential adverse effects of indole-3-propionic acid, resulting in an overall beneficial outcome. Alternatively, the elevated concentration of indole-3-propionic acid detected in individuals with AD and PD may represent a potential response mechanism by the body to counteract the progression of these diseases by upregulating this tryptophan metabolite. Nevertheless, additional research has also demonstrated the anti-oxidative and anti-inflammatory properties of indoles, resulting in decreased neurodegeneration (Yin et al., 2021, 2023). These findings underscore the potential therapeutic advantage of these metabolites (Pappolla et al., 2021; Zhou et al., 2023).

Overall, exploring bacterial proteins and metabolites in the context of neurodegeneration has opened a fascinating avenue of research that underscores the intricate relationship between the gut and brain. Microbial proteins, such as curli and gingipain, and specific metabolites, like SCFAs, p-cresol, indoles, and protein-bound UTs have been implicated in influencing neuroinflammation, oxidative stress, and protein aggregation. The precise mechanisms are still evolving and occasionally contradictory; however, these findings shed light on the complexity of the GBA and its significance in neurodegenerative diseases like AD and PD. As we continue to explore the connection between the gut microbiome and neurological health, there is a growing realisation that these bacterial proteins and metabolites may be pivotal in devising innovative therapeutic strategies and preventive measures against the debilitating effects of neurodegeneration. Novel technologies and applications such as metagenomics and metaproteomics offer immense promise and will be indispensable tools in unravelling the complex interplay between the gut microbiota and neurodegenerative conditions.

## 4. Psychiatric disorders in AD and PD

Alongside gut dysbiosis, psychiatric disorders, such as depression and anxiety, often accompany AD and PD, adding complexity to the management of these neurodegenerative diseases (Lin et al., 2015; Santabarbara et al., 2019). Individuals with psychiatric conditions are at four times greater risk of developing a neurodegenerative disease (Wingo et al., 2022). Furthermore, approximately 65% of patients with neurodegenerative diseases experience psychiatric symptoms (Van Der Linde et al., 2016). In AD, depression and anxiety are common psychiatric symptoms that arise at different disease stages. Similarly, in PD, depression and anxiety are prevalent non-motor symptoms that can significantly affect the overall mental health of patients. A meta-analysis of individuals with anxiety revealed an increased risk of dementia later in life, confirming the findings of several other studies (Gulpers et al., 2016; Gimson et al., 2018; Santabarbara et al., 2020). One study indicated that the correlation between anxiety and the onset of dementia was stronger in those who developed anxiety in later years, suggesting that anxiety is a possible indicator of dementia development (Gulpers et al., 2016). Evidence of an association between anxiety and PD has shown similar results. Anxiety manifests more than 25 years prior to the onset of motor symptoms in a subset of patients with PD, highlighting anxiety as a possible indicator of PD development (Seritan et al., 2019). Another study showed that this risk increases with anxiety severity (Lin et al., 2015).

The mechanisms underlying the relationship between neurodegenerative diseases and psychiatric symptoms are complex and multifactorial. Neurochemical imbalances, structural brain changes, and the psychosocial impact of living with these chronic conditions contribute to the development and exacerbation of depression and anxiety. Recognising and addressing these mental health challenges in patients with AD and PD is crucial for providing comprehensive care and improving their overall quality of life.

One study investigated the common underlying mechanisms of major psychiatric and neurodegenerative diseases (Wingo et al., 2022). Psychiatric disorders included major depressive disorder, anxiety disorders, bipolar disorder, schizophrenia, post-traumatic stress, and alcohol abuse. Neurodegenerative diseases in this study included AD, PD, Lewy body dementia, amyotrophic lateral sclerosis, and frontotemporal dementia. The authors identified 13 shared causal proteins between major psychiatric and neurodegenerative diseases and several biological processes, including vesicular transport, synaptic transmission, immune function, and mitochondrial processes, by combining data from genome-wide association study results with human brain transcriptomes and proteomes. The discovery of 13 causal proteins shared between psychiatric and neurodegenerative disorders was an interesting finding. Further research is essential to validate these findings and explore the potential of these 13 proteins as targets for therapeutic purposes.

Importantly, in the context of neurodegenerative diseases, psychiatric disorders should be considered in conjunction with gut dysbiosis as these two prodromal features of neurodegeneration are not mutually exclusive (Berg et al., 2015; Ehrenberg et al., 2018; Huang et al., 2023; Jemimah et al., 2023). In fact, many patients with psychiatric disorders report symptoms of gut dysbiosis (Rogers et al., 2016). Given the evidence discussed above suggesting that the gut microbiome may impact brain function and behavior, and influence the development of neurodegenerative diseases, it is not surprising that the same could be true for psychiatric disorders. Our growing understanding that alterations in the gut microbiome can have downstream effects on brain function highlights the complex and multifaceted interactions of the gut microbiome (Mohajeri et al., 2018; Zhu et al., 2020). Longitudinal studies tracking neurodegenerative disease progression, ranging from the healthy, undiagnosed stage, to the final stages of the disease, are imperative if we are to gain a comprehensive understanding of the intricate relationship between neurodegeneration, gut dysbiosis and psychiatric disorders. In particular, whether gut dysbiosis and/or psychiatric disorders are causal of neurodegeneration, and therefore a modifiable risk factor, or simply a prodromal feature of neurodegenerative diseases, will be indispensable in directing the development of preventive and therapeutic approaches.

## 5. Current therapeutic approaches

Treatments for neurodegenerative diseases typically aim to boost specific neurotransmitters in the CNS either by increasing the substrate for neurotransmitter production or inhibiting neurotransmitter clearance mechanisms (Cacabelos, 2017; Cummings et al., 2019). These pharmacological treatments have enormous benefits for patients through symptom management; however, most of these drugs do not alter the disease course. Two drugs, aducanumab and lecanemab, aimed at modifying the progression of AD were approved by the FDA in 2021 and 2023, respectively, despite recommendations against approval of aducanumab by the FDA advisory panel (Dhillon, 2021; Van Dyck et al., 2023). The decision to approve lecanemab did not involve an advisory panel and the two drugs were approved under the accelerated FDA approval pathway, meaning that data from phase III clinical trials were not considered when deciding approval. Evidence for the efficacy of aducanumab is limited, with only one of the two phase III trials demonstrating positive outcomes (Budd Haeberlein et al., 2022). Although the efficacy of lecanemab is greater than aducanumab, there are safety concerns, with several reported deaths suspected to have resulted from amyloid-related imaging abnormalities (Reardon, 2023; Van Dyck et al., 2023). Trials are ongoing to validate the safety and efficacy for both drugs (Verger et al., 2023).

Given the underwhelming results of clinical trials over the last several decades, there is a pressing need to identify alternative treatment and prevention strategies, in addition to pharmacological treatment, as the number of people afflicted with neurodegenerative diseases increases (Liu et al., 2019; Kim et al., 2022; Mari and Mestre, 2022). One approach that is being heavily explored is the identification of biomarkers to detect the disease prior to onset or in the early stages to minimise neuronal loss and symptom severity. Several types of biomarkers are being investigated, including imaging biomarkers such as PET scans, CSF biomarkers and more recently, blood-based biomarkers (Bellaver et al., 2021, 2023; Katayama et al., 2021; Klatt et al., 2021; Mila-Aloma et al., 2022; De Picker et al., 2023; Gonzalez-Ortiz et al., 2023; Hazan et al., 2023; Lancini et al., 2023; Qi et al., 2023; Qu et al., 2023; Zhang J. et al., 2023). Blood-based biomarkers hold great potential as a non-invasive diagnostic tool as well as monitoring disease progression during treatment. A recently published paper in *The Lancet Neurology* marks a breakthrough in PD research (Siderowf et al., 2023). The paper describes a new assay which is able to detect pathological α-syn in the CSF of patients already diagnosed with PD and those who are at high risk but do not yet display symptoms. For extensive reviews on biomarkers in AD and PD, please refer to the following reviews (Li and Le, 2020; Klyucherev et al., 2022). Ongoing research continues to refine and validate biomarkers, paving the way for improved clinical management and the development of targeted treatments. Nevertheless, disease prevention necessitates a continued and urgent pursuit in understanding the molecular mechanisms involved in disease pathogenesis.

## 6. Alternatives to pharmacological treatment

### 6.1. Diet

Acknowledging the involvement of the gut microbiome and GBA in AD and PD has spurred new research endeavors to explore the potential contribution of diet in the pathogenesis of these diseases. Compared with healthy individuals, those with neurodegenerative diseases exhibit distinct changes in their gut microbiome (Romano et al., 2021; Chandra et al., 2023). Reduced SCFA’s in PD has led researchers to explore supplementation of dietary fiber to boost SCFA levels, a metabolite linked to neuroprotection in individuals with PD (Baert et al., 2021). Indole, and subsequently indole sulphate, is derived from the metabolism of tryptophan. Tryptophan is present in low amounts in plants compared to animal products (Li et al., 2021). Given the potential role of indoxyl sulphate in neurodegeneration, dietary changes to include more plant foods and less animal products may prove beneficial. In fact, Mediterranean and vegan diets have been associated with a lower risk of AD and PD, possibly owing to their high fiber and low saturated fat profiles (McCarty and Lerner, 2021; Katonova et al., 2022; Stefaniak et al., 2022). In fact, a Western diet, characterised by foods high in saturated fat, salt, refined sugar, and low in fiber with an overall high energy intake, has been linked to neurodegeneration, including AD and PD (Qu et al., 2019; Hong et al., 2021). Moreover, a Western diet increases systemic inflammation and neuroinflammation, impairs the BBB, and exacerbates protein pathologies, all of which are common features of neurodegenerative diseases (Wieckowska-Gacek et al., 2021a,b). Hence, studies have focused on identifying factors influencing this alteration and whether dietary modifications can restore gut microbiome homeostasis and brain health.

Besides lowering the incidence of neurodegenerative diseases, strict diets have demonstrated benefits in the symptom management of patients with AD and PD. An ovo-lacto vegetarian diet, defined as a type of vegetarian diet that excludes meat, poultry and seafood but includes both eggs (ovo) and dairy products (lacto), combined with bowel cleansing was shown to reduce motor symptoms in individuals with PD (Hegelmaier et al., 2020). Whereas a Mediterranean diet was associated with improved cognitive performance in individuals with dementia and mild cognitive impairment (Anastasiou et al., 2017). Given the promising results observed in individuals with AD and PD from dietary interventions, numerous clinical trials are underway, or recently completed, to investigate the effect of various diets, such as gluten-free (NCT05238545), ketogenic (NCT04701957), and Mediterranean diets (NCT04683900), in managing symptoms.

Dietary modification is a plausible avenue for treating and preventing neurodegenerative diseases. As our understanding of the intricate relationship between nutrition and brain health deepens, research continues to unveil promising strategies for managing symptoms and reducing the risk of conditions such as AD and PD. Nevertheless, further investigation is needed to refine dietary recommendations, and pharmacological intervention remains the primary strategy in treating neurodegenerative diseases.

### 6.2. Probiotics and FMT

Probiotics and FMTs have gained attention as potential therapeutic strategies to modulate the microbiome of patients with AD and PD, alleviating symptom severity. These strategies have been reviewed in detail (Sun and Shen, 2018; Varesi et al., 2022). Probiotics are live microorganisms, often referred to as “friendly bacteria,” which can provide health benefits when consumed in adequate amounts (Gomaa, 2020). Probiotics are also under investigation for their potential to support mental well-being through enhancing gut health (Sharma and Bajwa, 2021). This aspect is particularly relevant since individuals dealing with gut issues and neurodegenerative conditions often experience challenges related to their mental health (Zhang et al., 2022a; Intili et al., 2023). Probiotics aimed at enhancing the gut and brain health together are referred to as psychobiotics and are being investigated in neurodegenerative and neuropsychiatric disorders (Sharma and Bajwa, 2021). Psychobiotics, have been shown to ameliorate symptoms in neurodegenerative diseases like AD and PD and neuropsychiatric diseases such as anxiety and depression which are discussed in detail in these reviews (Sharma and Bajwa, 2021; Barrio et al., 2022). Psychobiotics can help to restore a balanced gut microbiota composition, potentially mitigating neuroinflammation and oxidative stress and may support gut barrier integrity, reducing the risk of harmful substances entering the bloodstream and affecting the brain (Kesika et al., 2021; Naomi et al., 2021; Wang Q. et al., 2021; Mirzaei et al., 2022). Studies investigating the therapeutic potential of probiotics in treating neurodegenerative disease have yielded promising outcomes, with results demonstrating amelioration of cognitive deficits and motor dysfunctions in rat and mouse models of AD and PD, respectively, alongside reductions in protein aggregation (Nimgampalle and Kuna, 2017; Hsieh et al., 2020). Psychobiotic supplementation has also been investigated as a preventive strategy to mitigate or delay neurodegeneration, with positive outcomes demonstrated in AD mouse models (Huang H. J. et al., 2021; Abdelhamid et al., 2022). Encouraging results of psychobiotic treatment in animal models of AD and PD have led to many currently active clinical trials (including NCT05145881, NCT06019117, NCT03968133, and NCT05146921). The efficacy of probiotics in these clinical trials will be determined based on outcome measures such as cognitive ability, motor function, neuropsychiatric symptoms, and general gut health. Clinical trials, such as those listed above, will help establish the safety and efficacy of psychobiotics, whether employed as a standalone therapeutic approach or as a complementary treatment for neurodegenerative diseases. Overall, research suggests that these psychobiotics may have the potential to alleviate symptoms of mood disorders like depression and anxiety, as well as improve symptoms in neurodegenerative diseases like AD and PD. While the field is relatively young, psychobiotics offer promising insights into the intricate connection between our gut and mental health, paving the way for innovative approaches to improving well-being through the manipulation of the microbiome.

An FMT involves the transplantation of faecal material from a healthy donor into the gut of a recipient. This approach aims to restore a healthier gut microbiota by introducing a diverse and balanced microbial community (Khoruts and Sadowsky, 2016). In an AD mouse model, an FMT resulted in improved cognitive abilities and reduced pathological protein levels (Sun et al., 2019). Similar results were observed in an MPTP-induced mouse model of PD, where motor function improved and markers of inflammation were reduced following an FMT (Sun et al., 2018). These studies using animal models were followed by a recent pilot study in patients with PD, demonstrating increased gut microbiome diversity following multiple FMTs. Patients also reported improved motor functions. However, this study was limited by its relatively small sample size and limited follow-up of 9 months (DuPont et al., 2023). Studies with larger cohorts and longer follow-up periods are crucial to understand the safety, efficacy, and long-term effects of FMTs in AD and PD. The advantages of FMTs lie in their potential to introduce a more significant and comprehensive microbial diversity into the patient’s gut, which may positively affect neuroinflammation and other pathological processes. However, this method is still in its infancy in neurodegenerative diseases, and there are several challenges and concerns. One major challenge is ensuring the safety and standardisation of FMT procedures. Ensuring that donors are disease-free and transplant material is properly screened and processed is crucial to avoid potential complications. Moreover, the individual variability in gut microbiota and the complexity of neurodegenerative diseases means that not all patients may respond the same way to probiotics or FMTs. Personalised approaches may be required to tailor these therapies to specific patient profiles. Utilising metagenomics and metaproteomics, clinicians can gain insights into the diversity of a patient’s microbiome and design a personalised treatment plan accordingly (Zmora et al., 2016; Kashyap et al., 2017). Next-generation probiotics encompass a larger diversity of organisms than traditional probiotics, encouraging the development of personalised probiotics. These next-generation probiotics possess advantages over traditional probiotics and FMTs, as their composition can be tailored to suit the specific needs of each individual (O’Toole et al., 2017; Zhang et al., 2022b). However, a considerable amount of research is still needed to understand how and what changes in the gut microbiome may confer risk to disease development before such a personalised approach becomes a reality. In contrast to probiotics, which are ingested, FMTs can be directly injected into the colon, bypassing the acidic stomach environment. This must be considered in the development and use of probiotics (Ojha et al., 2023). Despite limitations to probiotics and FMTs, these therapeutic strategies hold promise, but their clinical applications and long-term effects must be carefully studied and refined. As our understanding of the gut-brain connection deepens, these approaches may become valuable tools in managing neurodegenerative diseases.

### 6.3. Exercise

Exercise is another lifestyle change that has received limited research attention, and its potential effects may be underestimated. Exercise cannot cure these diseases; however, it has numerous benefits in slowing their progression, improving symptoms, and enhancing the overall well-being of patients (Santiago and Potashkin, 2023). Furthermore, recent studies have investigated the protective role of exercise in preventing the development of neurodegenerative diseases such as AD and PD. A 2016 meta-analysis of prospective observational cohort studies examining the link between physical activity and AD onset revealed a protective effect of exercise in reducing the risk of developing AD (Santos-Lozano et al., 2016). Two recent studies, one conducted in Japan and another in Korea, demonstrated a reduced risk of developing dementia with moderate-to-vigorous and light-intensity exercises, respectively (Yoon et al., 2021; Ihira et al., 2022). Vigorous physical activity, even if performed once a week, can protect against several chronic diseases, including AD and PD (Marques et al., 2018). Although a range of exercise intensities can offer protective advantages against the development of neurodegenerative diseases, engaging in more rigorous physical activity might provide heightened protection against AD onset (Lopez-Ortiz et al., 2023). In addition to reducing the risk of neurodegenerative diseases, physical activity has neuroprotective, symptomatic, cognitive, emotional, and social benefits, making it an essential component of a comprehensive care plan for individuals living with these challenging conditions. A study conducted in an NSE/APPsw Tg mouse model of AD indicated that exercise reduced pathological protein levels and conferred neuronal protection more than sedentary controls (Um et al., 2008). Recent mouse studies have further underscored the therapeutic potential of exercise, specifically running, in AD management. These studies have hinted at potential mechanisms, including heightened microglial glucose metabolism and increased plaque clearance through improved ubiquitin or proteasome pathways (Mehla et al., 2022; Xu et al., 2022; Zhang et al., 2022).

Similar studies conducted in male PD patients revealed that moderate daily physical activity protects against PD development (Yang et al., 2015). The authors observed no beneficial effects of occupational physical activity on neurodegenerative diseases, consistent with previous findings (Yang et al., 2015; Shih et al., 2016). This observation is interesting and indicates the importance of a relaxing, stress-free environment in facilitating the positive effect of exercise on PD, emphasising the connection between mental well-being and neurodegenerative conditions. Animal studies in PD models have linked exercise to reduced inflammation, decreased α-syn levels, improved mitochondrial function, and increased neurotrophic and vascular endothelial growth factors (Crotty and Schwarzschild, 2020; Lopez-Ortiz et al., 2023). A meta-analysis was conducted to investigate the role of exercise as a supplementary therapy alongside standard drug treatments for PD (Choi et al., 2020). The analysis categorised studies based on the type of exercise interventions used. The results indicated a notable improvement in motor symptoms with exercise but no effect on non-motor symptoms, with the effectiveness of exercise hinging on the type employed. Based on their findings, they have proposed the type of exercise that is most beneficial to the desired outcome. Nevertheless, the type and intensity of exercise will likely be specific to an individual, based on specific needs and capabilities, rather than adhering strictly to a proven optimal approach. Although non-motor symptoms were not improved, this meta-analysis encompassed a relatively small sample size, involving only 1,144 patients with PD across 18 studies.

Although exercise can confer benefits in preventing and managing AD and PD, more rigorous studies involving larger cohorts and longer follow-up periods are required to understand the beneficial types and intensities of exercise and molecular pathways involved. Furthermore, exercise positively affects gut health and mental well-being, which are associated with neurodegeneration, highlighting how these diseases are interwoven (Chekroud et al., 2018; Gubert et al., 2020).

## 7. Conclusion

Neurodegenerative diseases present a significant and growing global health challenge. Their progressive nature, multifactorial causes, alongside an aging population, is expected to lead to substantial socioeconomic burdens. Recent research has highlighted the crucial role of the GBA in neurodegeneration. The gut microbiome, an essential component of the GBA, has emerged as a focal point of investigation due to its potential to influence brain function and behavior. Dysbiosis in the gut microbiome has been linked to AD and PD, impacting neuroinflammation, protein aggregation and neurotransmitter production. Moreover, psychiatric disorders, particularly depression and anxiety, frequently co-occur with neurodegenerative diseases, adding further complexity to their management.

Current pharmacological treatments for neurodegenerative diseases primarily focus on symptom management, with limited success in modifying disease progression. The development of reliable biomarkers for early diagnosis and targeted treatments represents a promising avenue for future research. Alternative approaches, such as dietary modifications and exercise, have shown potential in modulating the gut microbiome and influencing disease outcomes. Adopting a personalised approach in leveraging probiotics, FMTs, and tailored exercise regimes may offer additional benefits for patients with neurodegenerative diseases.

In summary, the interplay between the GBA and psychiatric disorders in the context of neurodegenerative diseases provides a comprehensive framework for exploring new preventive measures and therapeutic strategies. By adopting a holistic perspective and continuing to unravel the intricate connections in this triad of neurodegeneration, gut dysbiosis, and psychiatric disorders, we can strive to enhance the quality of life for affected individuals and make significant progress in understanding neurodegeneration.

## Author contributions

CD: Conceptualization, Writing – original draft, Writing – review & editing. SP: Writing – review & editing. JJ: Conceptualization, Funding acquisition, Writing – original draft, Writing – review & editing.
